# Hospital Coding of Postoperative Ileus: A Prospective Study

**DOI:** 10.7759/cureus.24946

**Published:** 2022-05-12

**Authors:** John W Cromwell, Laura W Lund

**Affiliations:** 1 Gastrointestinal Surgery, University of Iowa Hospitals and Clinics, Iowa City, USA; 2 Clinical Research, Clinical Research Strategies, Pittsburgh, USA

**Keywords:** gastrointestinal surgery, clinical coding, reimbursement, clinical documentation, surgery, gastroenterology, poi, postoperative ileus

## Abstract

Background

Postoperative ileus (POI) is among the most common complications affecting patients who undergo major abdominal surgery. Because of the high volume of major surgery and the high incidence of postoperative ileus, failure to code for this complication may have a significant impact on hospital reimbursement and quality measures.

Objectives

This paper investigates the magnitude of the difference between the prevalence of POI as coded in administrative data versus the prevalence based upon a prospectively applied operational definition of POI in patients undergoing intestinal resection surgery.

Methods

Data was collected during the course of a prospective study at the University of Iowa Hospitals & Clinics on an investigational digital health device for predicting operationally defined POI. Following the first 24 hours post-surgery, a patient was identified as experiencing POI as operationally defined by the occurrence of vomiting, reversal of diet and/or placement of a nasogastric tube. For all subjects, billing data was also collected.

Results

A total of 203 adult patients undergoing intestinal resection surgery consented to participate. Of patients who developed POI based on the operational definition, 35% were not coded accordingly to capture appropriate risk adjustment and reimbursement.

Conclusions

Patients who experienced indicators of POI but who were not coded experienced over two days of additional time in the hospital compared to patients who did not experience POI, representing significant unreimbursed costs. Timing and duration of POI indicators appear to impact coding discrepancies and may suggest means for improving caregiver identification of POI in a patient’s medical record.

## Introduction

Hospital coding is a critical activity that underlies hospital billing, reimbursement, performance-based financial penalties, quality benchmarking, and consumer comparison. Thus, inaccuracy in coding may lead to broad and highly visible downstream negative impacts for hospitals. Postoperative ileus (POI) is a known complication of major abdominal surgery that is manifested by a profound paralysis of the gastrointestinal tract that may lead to increased length of stay, hospital readmission, requirement for parenteral feeding, aspiration pneumonia, dehydration, acute kidney injury, and additional morbidities [1,2]. Hospitalization for patients who develop POI has been shown to be both statistically longer and costlier compared to patients who do not develop postoperative POI, with differences that are clinically and financially meaningful [3,4]. Under the International Classification of Diseases, Tenth Revision, with Clinical Modifications, or ICD-10-CM coding system, POI is classified as a complication or comorbidity (CC), or major complication or comorbidity (MCC), by Centers for Medicare & Medicaid Services (CMS), increasing the relative weight assigned to the encounter. In the absence of other complications or comorbidities, appropriate coding of POI increases reimbursement and the overall hospital case mix index (CMI) to cover higher costs associated with POI patient care.

Following elective major abdominal surgery, patients commonly suffer from immediate postoperative nausea and vomiting (PONV) within the first 24 hours. PONV appears to be a centrally mediated entity due to the numerous effects of anesthetic agents. This is physiologically different from other causes of nausea and vomiting that occur later in the postoperative period (often 2-10 days later) which are most often caused by either functional or mechanical obstruction of the gastrointestinal tract, such as POI or early postoperative bowel obstruction. These are events that have a high likelihood of either resulting in the need to delay hospital discharge or may require hospital readmission if occurring following hospital discharge. Inadequate identification of POI by hospital caregivers in a patient’s medical record can result in a substantial loss of cost reimbursement [4-6]. The objective of this paper is to utilize prospectively and independently collected measures of POI from an investigational medical device study to evaluate coding accuracy.

## Materials and methods

Adult patients undergoing intestinal resection surgery at the University of Iowa Hospitals & Clinics were enrolled in a prospective clinical trial to develop a noninvasive digital health device designed to predict POI in this patient population (NCT03505476). The noninvasive investigational device adheres to the skin of the abdomen and captures intestinal sounds that can be used to provide a real-time prediction of POI. The study was approved by the University of Iowa Institutional Review Board and all patients provided written informed consent which included consent to collect billing data from their encounter. The primary objective of the study was to maximize the predictive value of the device and is not the subject of this paper; however, an exploratory objective of the study was the collection of billing data for future health economics analyses related to device efficacy. In this report, a post hoc analysis was conducted using the billing data compared to the prospectively collected clinical indicators of POI used to optimize the device.

Following surgery, each subject received standard postoperative care which included enhanced recovery measures consisting of early oral re-feeding, offered within the first 24 hours after completion of surgery. Data regarding clinical evidence of POI was prospectively gathered after the first 24 hours using a modification of the operational definition of POI developed by Asgeirsson et al. (2010) in a study aimed to assess the financial impact of POI for the 30-day episode of care for colectomy [5]. Subjects who exhibited any of the following beyond 24 hours from completion of surgery were classified as having POI: 1) vomiting, 2) reversal of diet to NPO (nothing by mouth), or 3) placement of a nasogastric (NG) tube. Study classification of POI was based on evidence of the defined criteria from the review of the subjects’ standard care medical records or by visual inspection during daily study visits while the subject remained in the hospital. The study's classification of POI was not entered into the subject’s medical records. The subject’s caregivers were blinded to the study POI classification data and to the device measures; therefore, the subject's medical records reflected standard information equivalent to a non-study patient. As this was not an interventional study, X-ray imaging was not utilized as a requirement to confirm the study's classification of POI; imaging is only utilized in our standard practice when it would impact the course of care. Subsequent coding of the subject’s charts followed standard institutional procedure.

Following discharge from the hospital and after the final coding of charts was completed, the Diagnosis Related Group (DRG) and ICD10 coding assignments for the encounter were queried. A single CC- or major concurrent comorbidity (MCC)-qualifying diagnosis is required to change a DRG assignment to one with a higher relative weight. We sought to determine whether a missing ICD10 for POI, when it was clinically present, would have impacted the final DRG assignment by examining all ICD10 codes for each encounter and whether each of these codes would qualify as either a CC or MCC. Additionally, we investigated characteristics of the study data that may illuminate why caregiver indication of POI may or may not have been reflected in the medical records for subsequent coding.

Statistical significance was determined by the multiple comparison of means method offered by one-way ANOVA, incorporating the Dunnett T3 post-hoc power analysis for control of Type 1 error [7], using SPSS® Statistics version 28.0.1.0 (IBM Corp, Armonk, USA) (* represents p-value less than 0.001).

## Results

Between April 24, 2018, and October 14, 2019, 203 subjects undergoing intestinal resection surgery were enrolled in the study. Cases included partial and total colectomy, and partial and complete proctectomy, with variations in additional procedural elements. In all cases, the ICD10 coding assignments and ileus classification were available for analysis, but study data for four subjects was not evaluable due to withdrawal or protocol deviations. Based on the operational definitions used for the study, 49 subjects (25%) exhibited clinical evidence of POI and 150 subjects did not. In those subjects with clinical evidence of postoperative ileus, 17 (35%) did not have a qualifying ICD10 code representing postoperative ileus. Other comorbidities or complications for 14 of these subjects resulted in CC or MCC codes being assigned and a corresponding increased DRG assignment, but three of the 17 encounters (17.6%) that did not have a qualifying ICD10 code would have been coded at a higher DRG level had one of the ICD10 codes for POI been present.

In subjects who did not exhibit any clinical evidence of POI and were not coded for a concurrent comorbidity (n = 150), the mean number of days of daily clinical assessment data for the study, which reflects time to hospital discharge after the 24-hour post-surgical window, was 3.9 (median of 4 days, range of 0 - 7). For subjects with study indicators of POI, the mean number of days with daily clinical assessments was 6.4 (median of 7 days, range 2 - 7). Table 1 summarizes study subjects for whom POI indicators were identified (POI+) and compares the durations and quantity of POI indicators in those that were coded for POI with those that were not. 

**Table 1:** Descriptive analysis of subjects with billing records obtained for comparison with study indicators of POI. *Daily clinical assessments for POI indicators began 24 hours after surgery until hospital discharge; **In patients with only 1 or 2 days of POI indicators; ^a^Compared to subjects with no measures of POI, p < 0.0001; ​​​​​​​^b^Compared to POI+ subjects who were coded, p = 0.01; ​​​​​​​^c^Compared to POI+ subjects who were coded, p < 0.001 POI: Postoperative ileus

	No POI Indicators Identified (POI-) (Not coded for POI)	POI Indicators Identified (POI+)	
		Coded for POI	Not Coded for POI
Total subjects with coding data (N = 199)	n = 150 (75% of Total)	n = 49 (25% of Total)	
		n = 32 (16% of Total) (65% of POI+)	n = 17 (9% of Total) (35% of POI+)
After first 24 hours from surgery	Mean (Median) [Range]		
Days with clinical assessments*	3.9 (4) [0 – 7]	6.4 (7) [2 – 7] ^a^	
		6.8 (7) [2 – 7] ^a^	5.6 (6) [3 – 7] ^a,b^
Days with one or more POI indicators	-	3.4 (3) [1 – 6]	1.5 (1) [1 – 5] ^c^
Days with multiple POI indicators	-	2.6 (2.5) [0 – 5]	0.5 (0) [0 – 5]^ c^
Days with vomiting	-	1.3 (1) [0 – 4]	0.9 (1) [0 – 2]
Days with NG tube	-	2.4 (3) [0 – 5]	0.4 (0) [ 0 – 5]^ c^
Days with Diet Reversal	-	2.9 (3) [0 – 5]	0.8 (0) [0 – 5]^ c^
First day where POI indicator occurred**	-	4.3 (4) [2 – 6] (n = 8)	2.5 (3) [1 – 4]^ c^ (n = 16)

**Figure 1:**
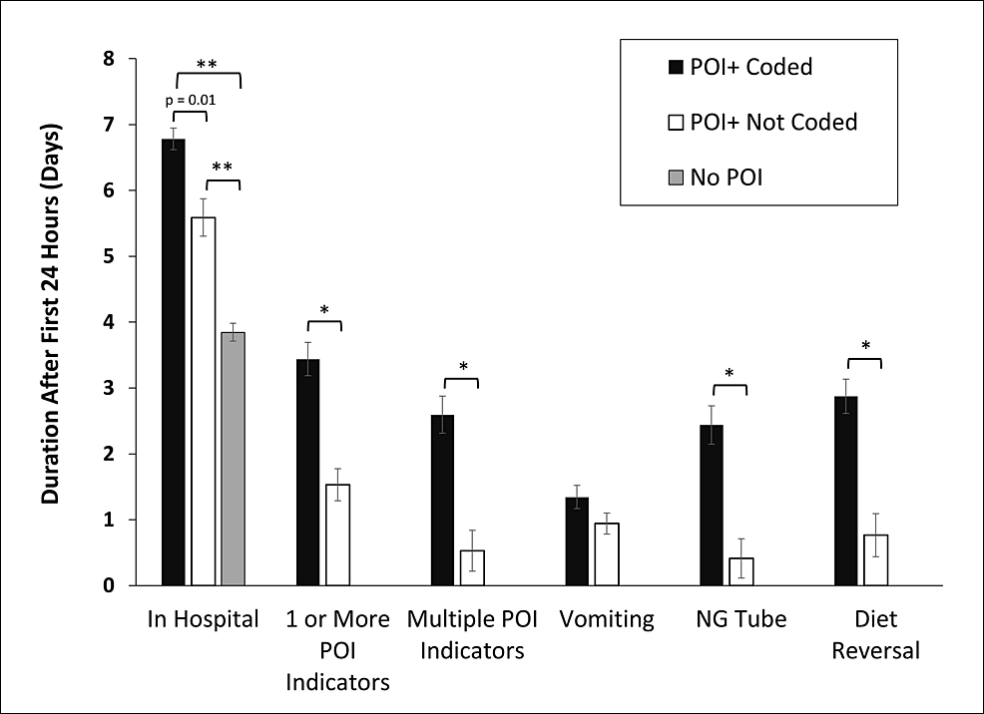
Comparison of study subjects with indicators of POI who were and were not coded for POI. Comparisons include the number of days after the first 24 hours post-surgery with one or more POI indicators, days with multiple POI indicators, days with vomiting, days with an NG tube and days with diet reversal (*p < 0.001, **p < 0.0001 based on Dunnett T3 post hoc power analysis for multiple comparison control of Type 1 error). POI: Postoperative ileus; NG: Nasogastric

Exploratory analyses were conducted to identify factors that might illuminate why caregivers of subjects with clinical indications of POI did not identify POI specifically in the medical charts for subsequent coding. Comparing POI+ subjects who were and were not coded for POI, there were significant differences in the number of days beyond the 24-hour period after surgery where the subject had one or more indicators of POI, and similarly, a significant difference in the number of days with multiple POI indicators (Figure 1). Uncoded POI+ subjects experienced fewer days with one or more POI indicators and with multiple POI indicators compared to coded POI+ subjects. For POI+ subjects with only one or two days of POI factors, the first day on which the factors were identified, beyond the first 24 hours, was sooner in uncoded patients compared to coded patients.

A possible relationship of coding to the three factors used in the study for identifying POI was also investigated. Uncoded subjects experienced significantly fewer days with an NG tube and with diet reversal but did not reflect significant differences in days with vomiting.

## Discussion

Postoperative ileus is among the most common complications of abdominal surgical procedures. The presence of POI is associated with substantially higher costs and duration of care. In 2007, Goldstein et al. estimated the total annual cost for hospitals to manage coded POI to be $1.46 billion, but also acknowledged that this likely under-represents the prevalence of POI due to the use of administrative data and lack of objective definitions for this condition [3]. The lack of concordance between coded POI and its actual prevalence may have substantial impact on hospitals by falsely lowering the case mix index and resulting in under-reimbursement in a DRG-based payment system.

In the current study, we sought to determine the magnitude of the difference between the prevalence of POI as coded in administrative data versus the prevalence based upon a prospectively applied operational definition of POI in patients undergoing colon resection surgery. Vather and others have pointed out the significant ambiguity in defining POI which has led to difficulties in comparing clinical studies [8]. Definitions that rely on features such as the delayed passage of stool or flatus may define an entity as having little operational consequence, unless leading to delays in either oral refeeding or hospital discharge. We chose to use a modification of Asgeirsson’s definition of POI that acknowledges those facets of POI requiring intervention or prolonged hospitalization, thus leading to increased costs of care. 25% of subjects in our study developed POI using this modified definition, which tracks closely with the 24% prevalence reported by Asgeirsson in their study [5].

Our finding that 35% of subjects with clinical evidence of POI lacked the presence of a respective ICD10 code in the final coding may have significant financial implications. At the time of this study, the base CMS reimbursement for MS-DRG 331 “Major Small & Large Bowel Procedures w/o CC/MCC” was $14,913.09. The base CMS reimbursement for MS-DRG 330 “Major Small & Large Bowel Procedures w CC” is $22,307.83. Thus, the marginal increase in CMS payment for each missed coding opportunity was $7,394.74. In this small study of 203 subjects, the total missed opportunity was $22,184.22.

To estimate the national economic magnitude of undercoded and unreimbursed POI, we considered Goldstein’s assumption of 142,026 coded encounters of POI in the U.S. annually for a selection of common abdominal procedures [3]. If the rate of undercoding reflected in our study is generalizable to these cases, then approximately 87,000 additional encounters for these procedures were undercoded for POI. This could grossly translate into a magnitude of nearly $100 million annually using CMS payment rules. Because POI is present in a far greater selection of surgeries, the actual value may be much higher.

Factors that may play a role in undercoding include when indicators of POI first occur, the duration of POI indicators and what factors are recognized as POI indicators. Patients who experienced POI indicators but were not coded experienced shorter duration and earlier appearance of indicators compared to coded patients. However, the number of hospital days in uncoded patients with POI indicators was 5.6 days compared to 3.9 days in patients who did not experience POI reflecting over two days of unreimbursed additional cost. While this analysis was ad hoc in nature and evaluated factors that are related, it suggests that standard institutional definitions of POI should be evaluated to improve caregiver charting for more effective coding and reimbursement.

A key strength of this study is that it uses a prospectively applied operational definition of POI to compare with final administrative ICD10 coding, the first such study that we are aware of to do so. While substantial undercoding of POI has been assumed in the literature [6], this is the first to directly demonstrate the magnitude of this undercoding in a homogenous group of colon resection encounters, and to identify potential factors that may help to rectify the discrepancy. This study also has significant limitations. Because it was performed in a single institution and in a homogenous cross-section of procedures, the generalizability of the findings is unknown. Additionally, the analysis of data was performed ad hoc and is exploratory in nature. There is also potential for readers to disagree with our choice of features for operationally defining POI, however, the prevalence of POI in our population closely resembles that of the original proponents of this definition. Additionally, this definition represents features that are known to increase the cost of care.

## Conclusions

Patients who experienced indicators of POI but who were not coded accordingly for reimbursement experienced over two days of additional time in the hospital compared to patients who did not experience POI, representing potentially significant unreimbursed costs. Use of the indicators of vomiting, NG tube, and/or diet reversal occurring after the first 24 hours post-surgery demonstrated effective representation of increased length of stay due to POI. Timing and duration of POI indicators appear to impact coding discrepancies and may suggest means for improving caregiver identification of POI in a patient’s medical record. Utilization of a systematic approach for identifying POI in medical records may reduce this discrepancy.

## References

[1] Venara A, Neunlist M, Slim K, Barbieux J, Colas PA, Hamy A, Meurette G (2016). Postoperative ileus: pathophysiology, incidence, and prevention. J Visc Surg.

[2] Bragg D, El-Sharkawy AM, Psaltis E, Maxwell-Armstrong CA, Lobo DN (2015). Postoperative ileus: recent developments in pathophysiology and management. Clin Nutr.

[3] Goldstein J, Matuszewski K, Delaney C (2007). Inpatient economic burden of postoperative ileus associated with abdominal surgery in the United States. P & T.

[4] Mao H, Milne TG, O'Grady G, Vather R, Edlin R, Bissett I (2019). Prolonged postoperative ileus significantly increases the cost of inpatient stay for patients undergoing elective colorectal surgery: results of a multivariate analysis of prospective data at a single institution. Dis Colon Rectum.

[5] Asgeirsson T, El-Badawi KI, Mahmood A, Barletta J, Luchtefeld M, Senagore AJ (2010). Postoperative ileus: it costs more than you expect. J Am Coll Surg.

[6] Iyer S, Saunders WB, Stemkowski S (2009). Economic burden of postoperative ileus associated with colectomy in the United States. J Manag Care Pharm.

[7] Sauder DC, Demars CE (2019). An updated recommendation for multiple comparisons. Adv Methods Pract Psychol Sci.

[8] Vather R, Trivedi S, Bissett I (2013). Defining postoperative ileus: results of a systematic review and global survey. J Gastrointest Surg.

